# A novel donor–π–acceptor halochromic 2,6-distyrylnaphthalene chromophore: synthesis, photophysical properties and DFT studies†

**DOI:** 10.1039/d0ra08508a

**Published:** 2020-12-22

**Authors:** Farhad Panahi, Ali Mahmoodi, Sajjad Ghodrati, Fazlolah Eshghi

**Affiliations:** Chemistry Department, College of Sciences, Shiraz University Shiraz 71454 Iran Panahi@shirazu.ac.ir; Department of Polymer Engineering and Color Technology, Amirkabir University of Technology Tehran Iran

## Abstract

In this study a new 2,6-distyryl naphthalene [2-((4-((*E*)-2-(6-((*E*)-2,4-bis(methylsulfonyl)styryl)naphthalen-2-yl)vinyl)phenyl)(ethyl)amino)ethan-1-ol; ASDSN] was synthesized successfully using Heck chemistry as the main reaction. The ASDSN compound is a donor–pi–acceptor (D–π–A) conjugated system with amino as electron donating and sulfonyl as electron withdrawing groups. The UV-vis absorption of ASDSN was observed in the range of 403–417 nm with high molar extinction coefficients (*ε* = 15 300–56 200 M^−1^ cm^−1^) in some different solvents. This new fluorescent 2,6-distyryl naphthalene compound emits in the yellow region of the visible spectrum (557 nm) with Stokes shifts of 5930 cm^−1^. ASDSN is a pH-responsive fluorescence compound that shows yellow fluorescence in neutral form and blue fluorescence in the protonated form. A white light emission (WLE) for the chromophore was observed at pH = 3.0. The ASDSN chromophore presented a satisfactory white light quantum yield (*Φ*) of 13% which was desirable for producing white light emitting devices. Density functional theory (DFT) and time-dependent (TD)-DFT were applied to study structural and electronic properties of the chromophore.

## Abstract 2

A new pH-responsive fluorescence chromophore with white light emission at pH = 3 was synthesized which has high potential application in the preparation of high-performance lighting devices.

## Graphical abstract

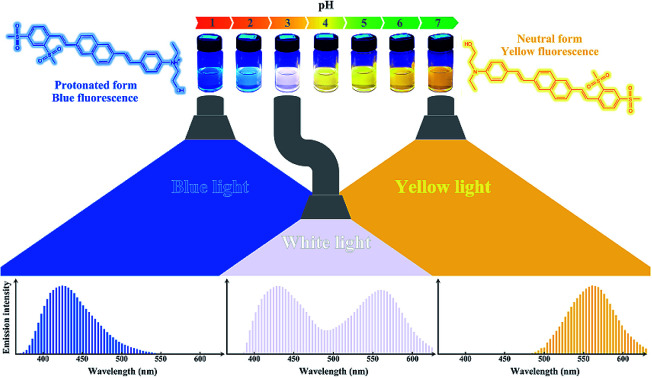

## Introduction

The synthesis of fluorophores incorporating distyrylbenzene π-conjugated systems has attracted considerable attention because of their high potential applications in optoelectronic and sensory devices as well as fluorescent probes in medical utilization and in organic light-emitting diodes.^1–6^ On the other hand, organic molecules that possess intramolecular charge transfer (ICT) character are important in a large number of areas, such as organic solar cells, polarity probes and nonlinear optics.^7,8^

Donor–π–acceptor (D–π–A; where D is an electron-donating group, A is an electron-accepting group, and π is a conjugating moiety) systems with different configurations have been extensively developed because their absorption spectra and energy gaps can be readily tuned by controlling the ICT character using different electron-donor and electron-acceptor groups. This belonging plays an important role in the improvement of the photophysical properties of photonic devices.^9–11^

Distyrylbenzenes are a well-known type of fluorescent dyes that possess high photochemical and thermal stability.^12,13^ To synthesize these conjugated spacers, two important organic transformations, namely Wittig and Heck reactions, are used. Depends on the availability of starting materials and the strategy used to prepare the stilbene and distyrylbenzene molecules, one of the aforementioned reactions is preferred to be used. While sometimes, a combination of both reactions is required to obtain desired stilbene and distyrylbenzene compounds. Typically, feeding compounds participating in the above mentioned reactions are already modified with electron donating and electron withdrawing substitutes.

The emission of distyrylbenzenes highly depends on the nature of electron donating and the electron-acceptor groups. With increasing the strength of the donor and/or the acceptor groups, frequently, a bathochromic shift is observable. So that, by use of suitable electron-donating and the electron-acceptor groups, the ICT band can even appear in the range of near and far-infrared.^14,15^ Sulfone (–SO_2_R), is one of the most interesting electron-acceptor groups that features remarkable mobility of charge carriers which is an important photophysical property in π-conjugated systems. In addition, hydrophobic rigid backbone of sulfone group with excellent charge generation property can facilitate the electron migration in the structure of molecule, which is another essential character in photonics.^16^ Amine functional groups are considered as compelling candidates to be used as efficient electron-donating moieties. The electron pair of amino groups can act as a ligand or base to coordinate with metals and abstract protons, respectively. Consequently, the amino-functionalized stilbene-based fluorescent materials are examined as potential sensors for detecting metal ions.^17–19^ Moreover, substantial shifts in the absorption and emission of amino-functionalized stilbenes are observed upon protonation, representing pH-sensory capability of these materials.^20^ In other words, they are halochromic chromophores with tunable fluorescence through regulation of pH. More interestingly, they can emit white fluorescence at specific pH, because the emission of the neutral and protonated forms of amino-functionalized stilbenes is different due to the presence of different molecule forms simultaneously. Therefore, the emission spectra cover the entire visible region ranging from 400 nm to 700 nm leading to white light emission (WLE).^21–28^ This exceptional feature enables white light emitter chromophores to be utilized in single-component white organic light-emitting diodes (WOLEDs).^29^

In this study, in continuation of our previous reports^13,30–33^ we synthesized a new D–π–A 2,6-distyryl naphthalene molecule containing an amino group as electron-donating and sulfonyl group as electron-withdrawing group in the terminals of the π-conjugated system. Two sulfonyl groups were used in *ortho* and *para* positions of the benzene ring in the acceptor terminal of the molecule in order to have a strong push–pull effect. We showed that the ASDSN chromophore experience a hypsochromic shift from yellow to blue with the variation of pH and emit white light at pH = 3. The chromophore presents a remarkable quantum yield (*Φ*) with the maximum value of 13 and 49% in its protonated and neutral state, respectively.

## Results and discussion

**Scheme 1:**
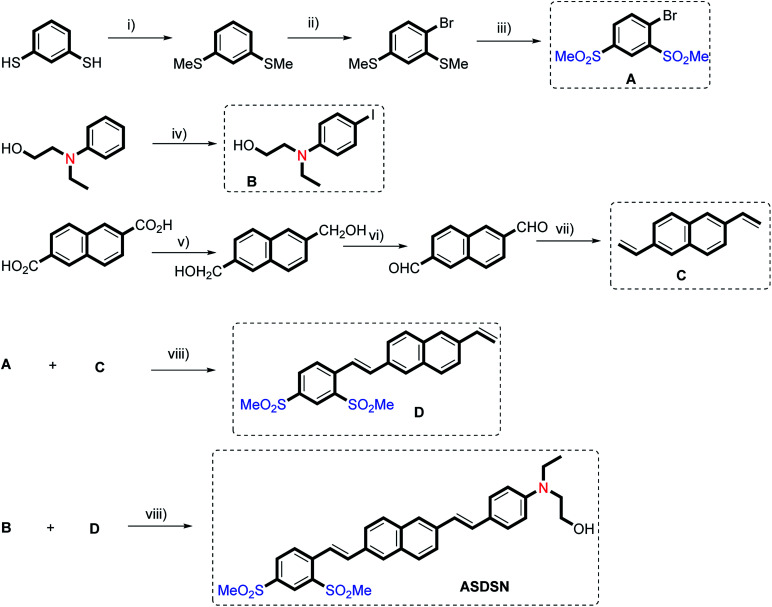
Synthetic routes to prepare compound ASDSN. (i) K_2_CO_3_, Me–I, acetone, 50 °C, 6 h. (ii) Bu_4_NBr (5 mol%), HNO_3_ (15% w/w), dichloroethane, 20 °C. (iii) AcOH, H_2_O_2_, CH_2_Cl_2_, 0 °C-reflux, 2 h. (iv) I_2_, dioxane/pyridine, 0 °C to rt, 3 h. (v) LiAlH_4_. (vi) PCC. (vii) Ph_3_PMeBr, KOtBu, THF, rt, 18 h. (viii) Pd(OAc)_2_ (1.2–1.8 mol%), bis[(2-diphenylphosphino)phenyl] ether (DPEPhos) (2.5–3.6 mol%), K_2_CO_3_, DMF, 120 °C, 6–12 h.

The Heck reaction using a palladium-catalyzed process was employed as a main organic transformation for the synthesis of the ASDSN chromophore (Scheme 1). The coupling partners in the Heck reaction are aryl halides and alkenes.^13^ Therefore, aryl halides A and B and also alkenes C and D were synthesized using available starting materials using organic transformations as shown in Scheme 1. To prepare the alkene intermediate D a controlled Heck reaction between aryl bromide A and alkene C was accomplished. Finally, the coupling reaction of alkene D and aryl iodide B afforded compound ASDSN with 87% isolated yield as a red solid.

**Fig. 1:**
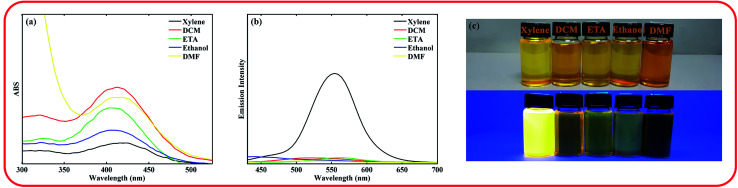
UV-vis spectra (a) and emission spectra (b) of ASDSN chromophore at concentration of 10^−5^ M in different solvents. The photographs of the chromophore in different solutions [from left to right, xylene, dichloromethane (DCM), ethyl acetate (ETA), ethanol, and dimethyl formamide (DMF)] were taken under natural daylight simulator (D65) lamps (top image), and irradiation of A-Class UV lamps (bottom image) (c).

After synthesis and characterization of the ASDSN molecule, its photophysical properties were investigated. The absorption and emission spectra of the chromophore in different solvents from low to high polarity were obtained to study the effect of solvent polarity on the photophysical behavior of the chromophore. The obtained results are shown in Fig. 1 and Table 1.

**Table 1:** Optical data of the ASDSN chromophore

Solvent	*λ*_ab_ (nm)	*λ*_em_ (nm)	Stockes shift (cm^−1^)	*ε* (L M^−1^ cm^−1^)	*E* a (eV)	*Φ* b
Xylene	417	557	5930	15 300	2.61	0.49
ETA	407	—	—	41 100	2.67	—
DCM	414	—	—	56 200	2.58	—
Ethanol	408	—	—	24 700	2.54	—
DMF	403	—	—	49 000	2.53	—

a Calculated from the absorption spectra by using the empirical formula of *E* (eV) = *hc*/*λ*_onset_ = 1240 (eV nm)/*λ*_max_ (nm).

b 9,10-Diphenylanthracene was used as standard (*Φ* = 0.90 in cyclohexane).

The ASDSN chromophore showed a broad and structure-less absorption band around 400 nm attributed to the ICT transition arising from the push–pull effect of the donor (amine) and the acceptor groups (sulfonyl). The absorption band of ASDSN chromophore experienced a minor change with the variation of solvent polarity that is related to the low dipole moment of the ground state of ASDSN. ASDSN was found to be fluorescence only in xylene with an emission band of 557 nm as well as an excellent fluorescence quantum yield (*Φ*) of 0.49. However, its fluorescence was quenched in more polar solvents. The observed strong fluorescence quenching in more polar solvents may be attributed to the non-radiative relaxation process. In other words, polar solvents can stabilize the excited state of the chromophore, decrease the energy level between the ground and excited state, and promote radiation less decay.^34–36^

**Fig. 2:**
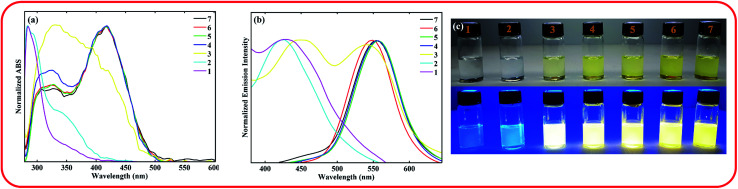
UV-vis spectra (a) and emission spectra (b) of the ASDSN chromophore in xylene at concentration of 10^−5^ M upon adjustment of pH from 1 to 7 by TFA. The photographs of the solutions (pH increases from left to right) were taken under natural daylight simulator (D65) lamps (top image), and irradiation of A-Class UV lamps (bottom image) (c).

The absorption and emission spectra of the compound in xylene containing various amounts of trifluoroacetic acid (TFA) were recorded to investigate the effect of pH on its photophysical. The obtained results are shown in Fig. 2.

**Fig. 3:**
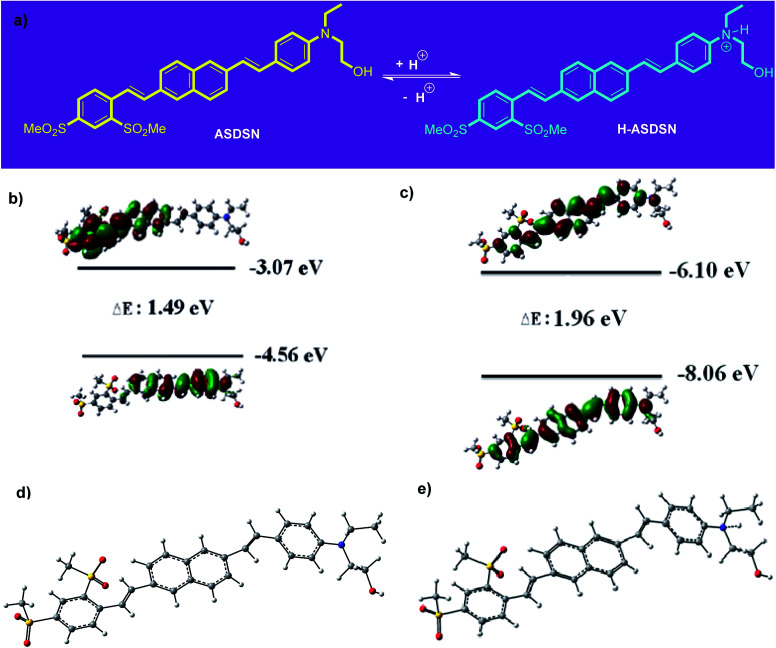
Protonation of ASDSN and formation of H-ASDSN form and their equilibrium in acidic environment (a). The HOMO–LUMO shapes of ASDSN and H-ASDSN and calculated energy gaps using DFT (b and c). Optimized structures of ASDSN (d) and H-ASDSN (e) using PBE/6-311++g (d, p) level of theory.

As shown in Fig. 2, with the decrease of pH from 7 to 4, no significant shift was observed for both absorption and emission bands of the chromophore. Further decreasing of pH from 4 to 1 caused a significant blue shift in the absorption and emission bands of the ASDSN chromophore. The color of the solution containing the ASDSN chromophore was changed from yellow to colorless, and its fluorescence color was changed from yellow to blue. The blue shift values for the chromophore were found to be 121 nm (from 405 to 284 nm) and 132 nm (from 554 to 422 nm) for the absorption and emission bands, respectively, when pH was changed from 7 to 1. These results can be attributed to the protonation of the chromophore by the addition of TFA, which decreases the ICT character of the chromophore (Fig. 3a). In general, amine groups, as a strong electron donor in the push–pull fluorescent molecule, are protonated in the presence of acids and converted to an electron acceptor (quaternary ammonium salt; H-ASDSN). Consequently, the lack of electron-donating ability in the structure of the molecule interrupts the push–pull effect of the chromophores and decreases its ICT character.^37–41^ The most striking result to emerge from the data shown in Fig. 2 was the broad emission for the chromophore at pH = 3, which resulted in almost pure white fluorescence with the commission international de l'eclairage (CIE 1931) color coordinates of (0.33, 0.32). This observation indicated that at specific pH, neutral and protonated form of the chromophore whose emission bands are complementary with a broad distribution could simultaneously exist in the solution and cause in white light emission.^42–45^ Another surprising outcome that emerged from the data was a noticeable white light quantum yield (*Φ*) of 0.13. This finding represented the great potential of this chromophore for white lighting device fabrication.

A DFT study was accomplished to have the frontier molecular orbitals of ASDSN and H-ASDSN [the highest occupied molecular orbital (HOMO) and lowest unoccupied molecular orbital (LUMO) orbitals] (Fig. 3b). The shape of orbitals shows that the HOMO and LUMO of ASDSN are both more centralized on donor (amino) and acceptor (sulfone) groups, respectively. While, H-ASDSN HOMO and LUMO orbitals are mostly centralized on π-conjugated system. The optimized geometry structure of both ASDSN and H-ASDSN are shown in Fig. 3d and e, respectively. It seems that the geometry of ASDSN molecule after protonation (in the form of H-ASDSN) not changed remarkably.

The interaction between ASDSN and solvents cause changing energy levels and re-distribution of electrons, excited state, dipole moment of the fluorophore, and substantial Stocks shift.^42,43^ These issues describe by Lippert–Mataga (LM) equation:1
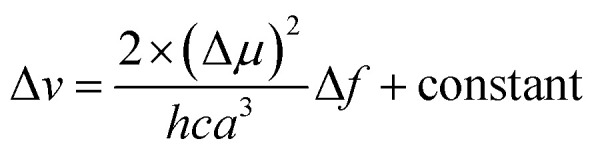
where Δ*ν*, Δ*μ*, *h*, *c*, *a* and Δ*f* are Stocks shift, difference of dipole moments, Planks constant, speed of light, radius of cavity, and solvent properties (below equation), respectively.2
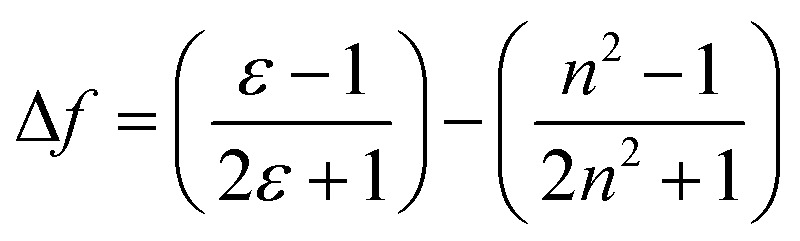


This equation shows the re-orientation of solvent molecules with dielectric constant (*ε*) and refractive index (*n*). The used solvents as different environments mainly result in change of dipole moment of these π-conjugated molecules (Table 2).

**Table 2:** Calculated stocks shift (cm^−1^) and re-orientation of solvent molecules (Δ*f*) in different solvents

Solvents	*ν*_A_ a	*ν*_E_ a	Δ*ν*	*ε*	*n*	Δ*f*
*para*-Xylene	19 263	16 418	2845	2.2705	1.4958	0.0033
ETA	19 415	16 356	3059	2.3832	1.4960	0.0138
DCM	19 924	16 552	3372	8.9300	1.4244	0.2170
Ethanol	20 741	17 016	3725	24.8520	1.3617	0.2890
DMF	20 113	16 569	3544	37.2190	1.4305	0.2747

a Wave number of absorption and emission 
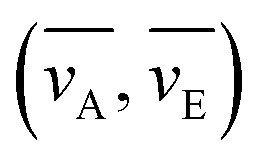
.

**Fig. 4:**
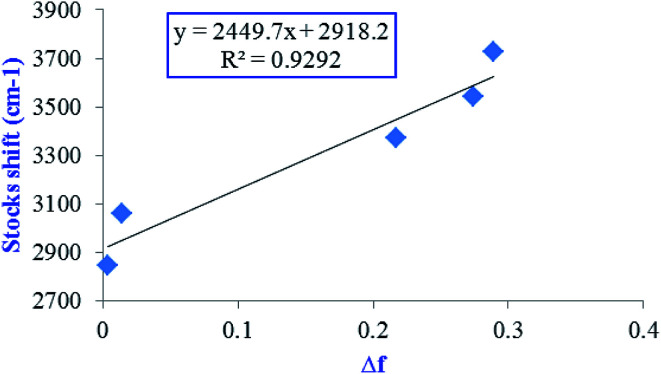
Lippert–Mataga plot for protic and aprotic solvents at selected level of theory.

In order to have Lippert–Mataga plot, the Stocks shift *vs.* environmental polarity was plotted (Fig. 4). High *R*^2^ index (0.93) in the Lippert–Mataga plot mainly confirms enhancing of dipole moment of excited state with increasing solvent polarity.

## Conclusion

In conclusion, a novel stilbene-based chromophore was synthesized through the Heck reaction. The compound showed a substantial hypsochromic shift from its neutral form with yellow fluorescence to its protonated form with blue emission. The chromophore displayed WLE at pH = 3 due to the simultaneous presence of its neutral and protonated forms representing pH-responsive fluorescence. Remarkable white light quantum yields of this fluorescent molecule either in its neutral or its protonated forms implied high potential application of this chromophore in the preparation of high-performance lighting devices. Theoretical study showed that the solvatochromic effect caused the changing of internal charge transfer (ICT) and dipole moment. Also, the protonation of luminophore mainly resulted in planarity and affected on the ASDSN properties.

## Experimental section

### General

Chemicals were purchased from Merck and Aldrich chemical companies. All the chemicals and solvents were used as received without further purification. For known products the spectral data were compared with those previously reported. For recording ^1^H NMR and ^13^C NMR spectra we used a Bruker (250 MHz) Avance DRX and samples were dissolved in pure deuterated CDCl_3_ with tetramethylsilane (TMS) as an internal standard. FT-IR spectroscopy (Shimadzu FT-IR 8300 spectrophotometer) was employed for characterization of the products. Melting points were determined in open capillary tubes in a Barnstead electro-thermal 9100 BZ circulating oil melting point apparatus. The reaction monitoring was accomplished by TLC on silica gel PolyGram SILG/UV254 plates. Column chromatography was carried out on columns of silica gel 60 (70–230 mesh).

### 1-Bromo-2,4-bis(methylsulfonyl)benzene (A)

In order to synthesis compound A, benzene-1,3-dithiol was used as available starting material. A 100 mL canonical flask was charged with benzene-1,3-dithiol (10 mmol, 1.42 g), K_2_CO_3_ (25 mmol, 3.45 g) and acetone (50 mL) at 0 °C. Then methyl iodide (22 mmol, 3.1 g) was added drop wise to the reaction mixture for 1 h. The reaction mixture was refluxed at 50 °C for 12 h. After completion of the reaction, the temperature was cool down to rt and the solvent was removed. The dense liquid was extracted with diethyl ether and the pure product was obtained by distillation (88%). ^1^H-NMR (250 MHz, DMSO-d_6_/TMS) *δ* (ppm): 2.41 (s, 6H), 7.52–7.96 (m, 3H). The spectral data was correspond to the previously reported method.^46^

For the bromination step, a 100 mL canonical flask was charged with KBr (20 mmol, 2.4 g), nitric acid (21% w/w, 40 mmol), dichloroethane (DCM, 20 mL), 1,3-bis(methylthio)benzene (10 mmol, 1.7 g), and tetrabutylammonium chloride (3 mol%, 0.9 g) and stirred at room temperature for 24 h. After the completion of the reaction, it was diluted with water (50 mL) and DCM (50 mL). The organic layer was separated from the aqueous layer and it was washed with saturated NaHCO_3_ and saturated NaCl. The organic layer was separated and dried over MgSO_4_. After concentration of organic layer under reduced pressure, the product was obtained as yellow oil (85%) which was used for next step without further purification.^47^

The obtained sulfide from previous step (2.0 g, 8 mmol) was dissolved in 15 mL of glacial acetic acid at 0 °C. Afterward, 30% hydrogen peroxide (6 mL) was added and it was refluxed for 2 h and then poured on crushed ice. Crud product was filtered, dried and purified by column chromatography using petroleum ether and ethyl acetate elunt to obtain pure product (A) as a white solid (2.0 g, 81%).^46^^1^H-NMR (250 MHz, CDCl_3_/TMS) *δ* (ppm): 3.65 (s, 6H), 7.48–7.95 (m, 3H). ^13^C-NMR (62.5 MHz, CDCl_3_/TMS) *δ* (ppm): 47.9, 48.1, 126.8, 127.2, 135.1, 135.3, 140.8, 143.9. Anal. cal. C_8_H_9_BrO_4_S_2_ (313.18): C, 30.68; H, 2.90; S, 20.47; found: C, 30.56; H, 2.81 0; S, 20.41.

### 2-(Ethyl(4-iodophenyl)amino)ethan-1-ol (B)

This compound was synthesized as a white solid based on a known procedure in the literature.^48^^1^H-NMR (250 MHz, CDCl_3_/TMS) *δ* (ppm): 1.16 (t, *J* = 7.0 Hz, 3H), 1.61 (brs, 1H), 3.35–3.43 (m, 4H), 3.75 (t, *J* = 5.5 Hz, 2H), 6.50 (d, *J* = 8.5 Hz, 2H), 7.42 (d, *J* = 8.5 Hz, 2H). ^13^C-NMR (62.5 MHz, CDCl_3_/TMS) *δ* (ppm): 11.7, 45.5, 52.4, 60.0, 114.9, 123.8, 137.7, 147.6. Anal. cal. C_10_H_14_INO (291.13): C, 41.26; H, 4.85; N, 4.81; found: C, 41.17; H, 4.78; N, 4.75.

### 2,6-Divinylnaphthalene (C)

This material was prepared in three steps using known procedures in the literature starting from naphthalene-2,6-dicarboxylic acid as commercially available chemical. First, naphthalene-2,6-dicarboxylic acid was converted to 2,6-bis(hydroxymethyl)naphthalene.^49^ A white solid was obtained which its spectral data completely matched to the previously reported one. ^1^H-NMR (250 MHz, CDCl_3_/TMS) *δ* (ppm): 4.11 (brs, 2H), 4.71 (d, *J* = 5.8 Hz, 2H) 7.47–7.50 (m, 2H), 7.79 (s, 2H), 7.81–7.84 (m, 2H). Anal. cal. C_12_H_12_O_2_ (188.23): C, 76.57; H, 6.43; found: C, 76.51; H, 6.34.

Next, 2,6-bis(hydroxymethyl)naphthalene was converted to naphthalene-2,6-dicarbaldehyde using an oxidation process by use of a known procedure.^50^^1^H-NMR (250 MHz, CDCl_3_/TMS) *δ* (ppm): 8.03–8.08 (m, 2H), 8.09–8.13 (m, 2H), 8.41 (s, 2H), 10.19 (s, 2H). Anal. cal. C_12_H_8_O_2_ (184.19): C, 78.25; H, 4.38; found: C, 78.18; H, 4.31.

Finally, naphthalene-2,6-dicarbaldehyde was transferred to 2,6-divinylnaphthalene (C) using a known procedure.^51^ A reaction vessel was charged with naphthalene-2,6-dicarbaldehyde (0.92 g, 5 mmol), KO*t*Bu (1.7 g, 15 mmol) and methyltriphenylphosphonium bromide (4.3 g, 12 mmol) and it was degassed and filled with argon. The reaction temperature kept at 0–5 °C and then dry THF (30 mL) was added and it was stirred for 1 h at this temperature. Afterward, the reaction mixture was allowed to stir at room temperature for another 6 h. After completion of the reaction it was concentrated, extracted (with ether and water) and dried over Na_2_SO_4_. The obtained organic layer was concentrated and purified by column chromatography (*n*-pentane) (72%, 0.65 g). ^1^H-NMR (250 MHz, CDCl_3_/TMS) *δ* (ppm): 5.25 (d, *J* = 10.9 Hz, 2H), 5.81 (d, *J* = 17.5 Hz, 2H), 6.81 (dd, *J* = 17.5, 10.6 Hz, 2H), 7.52–7.77 (m, 6H). Anal. cal. C_14_H_12_ (180.25): C, 93.29; H, 6.71; found: C, 93.21; H, 6.65.

### 2-1-2-[2-(2,4-Bis-methanesulfonyl-phenyl)-vinyl]-6-vinyl-naphthalene (D)

A sealed Schlenk tube was charged with 1-bromo-2,4-bis-methanesulfonyl-benzene (A; 1 mmol, 0.313 g), 2,6-divinyl-naphthalene (C; 1 mmol, 0.18 g), and K_2_CO_3_ (2.5 mmol, 0.345 g), Pd(OAc)_2_ (1.2 mol%, 2.7 mg), DPEPhos ligand (2.4 mol%, 13.0 mg) and it was evacuated and backfilled with argon. Then 5 mL of dry DMF was added to the reaction mixture under fellow of argon and tube was sealed with a screw-cap and the resulting mixture was heated in an oil bath at 120 °C for 6 h. The reaction was followed by TLC. After completion of the reaction, the mixture was cooled down to room temperature and filtered and the remaining solid was washed with dichloromethane (3 × 5 mL) in order to separate the catalyst. After the extraction of dichloromethane from water, the organic extract was dried over Na_2_SO_4_. The products were purified by column chromatography (hexane/ethyl acetate: 10/1) to obtain the pure product (0.31 g, 75%). Yellow solid; mp 213–215 °C. IR (KBr): 3749, 3441, 1851.5, 1419, 1304, 1165, 1134, 1103, 964, 818, 763, 509 cm^−1^. ^1^H-NMR (250 MHz, CDCl_3_/TMS) *δ* (ppm): 2.47 (s, 3H), 2.48 (s, 3H), 5.38 (d, *J* = 11.0 Hz, 1H), 6.01 (d, *J* = 17.5 Hz, 1H), 6.89 (dd, *J* = 17.6, 10.7 Hz, 1H), 7.68 (s, 1H), 7.75–7.85 (m, 3H), 7.92–8.01 (m, 4H), 8.08 (d, *J* = 3.2 Hz, 1H), 8.28–8.32 (m, 1H), 8.41 (d, *J* = 1.7 Hz, 1H). ^13^C-NMR (62.5 MHz, CDCl_3_/TMS) *δ* (ppm): 41.2, 42.0, 113.3, 128.5, 128.9, 129.0, 131.0, 131.6, 132.2, 132.3, 133.8, 134.0, 134.5, 135.6, 135.8, 139.2, 139.3. Anal. cal. C_22_H_20_O_4_S_2_ (412.5): C, 64.05; H, 4.89; O, 15.51; S, 15.55; found: C, 64.11; H, 4.93.

### 2-2-2-{[4-(2-{6-[2-(2,4-Bis-methanesulfonyl-phenyl)-vinyl]-naphthalen-2-yl}-vinyl)-phenyl]-ethyl-amino}-ethanol (ASDSN)

A sealed Schlenk tube was charged with compound D (0.5 mmol, 0.2 g), 2-[ethyl-(4-iodo-phenyl)-amino]-ethanol (B; 0.5 mmol, 0.14 g), and K_2_CO_3_ (2.0 mmol, 0.28 g), Pd(OAc)_2_ (1.2 mol%, 1.4 mg), DPEPhos ligand (2.4 mol%, 7.0 mg) and it was evacuated and backfilled with argon. Then 5 mL of dry DMF was added to the reaction mixture under fellow of argon and tube was sealed with a screw-cap and the resulting mixture was heated in an oil bath at 120 °C for 12 h. After completion of the reaction, the mixture was filtered (in hot form) and the remaining solid was washed with DMF (2 mL) in order to separate the catalyst. Afterward, water (10 mL) was added to the solution in order to precipitate product. The obtained solid was purified by column chromatography (hexane/ethyl acetate: 10/2) to obtain the pure product (0.25 g, 87%). Red solid; mp 289–291 °C. IR (KBr): 3441, 2924, 1589, 1520, 1304, 1165, 1142, 957, 802, 764, 656, 509 cm^−1^. ^1^H-NMR (250 MHz, DMSO-d_6_/TMS) *δ* (ppm): 1.10 (t, *J* = 4.5 Hz, 3H), 2.50 (t, *J* = 1.2 Hz, 2H), 1.10 (t, *J* = 4.5 Hz, 3H), 3.35 (s, 2H), 3.54–3.58 (m, 2H), 4.80 (t, *J* = 3.2 Hz, 1H), 6.70 (d, *J* = 5.5 Hz, 2H), 7.22 (dd, *J* = 49.5, 10.2 Hz, 2H), 7.46 (d, *J* = 5.5 Hz, 1H), 7.74 (d, *J* = 10.0 Hz, 1H), 7.82–7.87 (m, 2H), 7.92–7.95 (m, 3H), 8.03–8.08 (m, 2H), 8.23 (dd, *J* = 5.2, 1.2 Hz, 1H), 8.36 (d, *J* = 5.2 Hz, 1H), 8.43 (d, *J* = 1.2 Hz, 1H). ^13^C-NMR (62.5 MHz, CDCl_3_/TMS) *δ* (ppm): 12.0, 43.4, 43.8, 44.6, 52.0, 59.3, 111.3, 121.8, 122.6, 123.8, 123.9, 124.3, 127.9, 128.0, 128.3, 128.4, 128.6, 128.9, 130.0, 132.0, 133.8, 136.7, 136.9, 137.7, 139.3, 141.4, 147.6. Anal. cal. C_32_H_33_NO_5_S_2_ (575.7): C, 66.76; H, 5.78; N, 2.43; O, 13.89; S, 11.14; found: C, 66.74; H, 5.73; N, 2.47.

## Conflicts of interest

The authors declare no competing financial interests.

## Supplementary Material

RA-011-D0RA08508A-s001
