# Supplementary material for: A novel donor–π–acceptor halochromic 2,6-distyrylnaphthalene chromophore: synthesis, photophysical properties and DFT studies
Source: RSC Adv. 2020 Dec 22;11(1):168–76. doi: 10.1039/d0ra08508a (PMC8690040; doi:10.1039/d0ra08508a)
Supplement: RA-011-D0RA08508A-s001 [file RA-011-D0RA08508A-s001.pdf]

Electronic Supplementary Material (ESI) for RSC Advances.
This journal is © The Royal Society of Chemistry 2020

A Novel Donor-π-Acceptor Halochromic 2,6-Distyrylnaphthalene Chromophore: Synthesis, Photophysical Properties and DFT studies

Farhad Panahi,[a,*] Ali Mahmoodi,[b,] Sajjad Ghodrati,[b] Fazlolah Eshghi[a]

[a] Chemistry Department, College of Sciences, Shiraz University, Shiraz 71454, Iran

[b] Department of Polymer Engineering and Color Technology, Amirkabir University of Technology, Tehran, Iran

*Email: Panahi@shirazu.ac.ir

Copy of NMR spectra of synthesized compounds

^{1}H NMR of 2-[2-(2,4-Bis-methanesulfonyl-phenyl)-vinyl]-6-vinyl-naphthalene

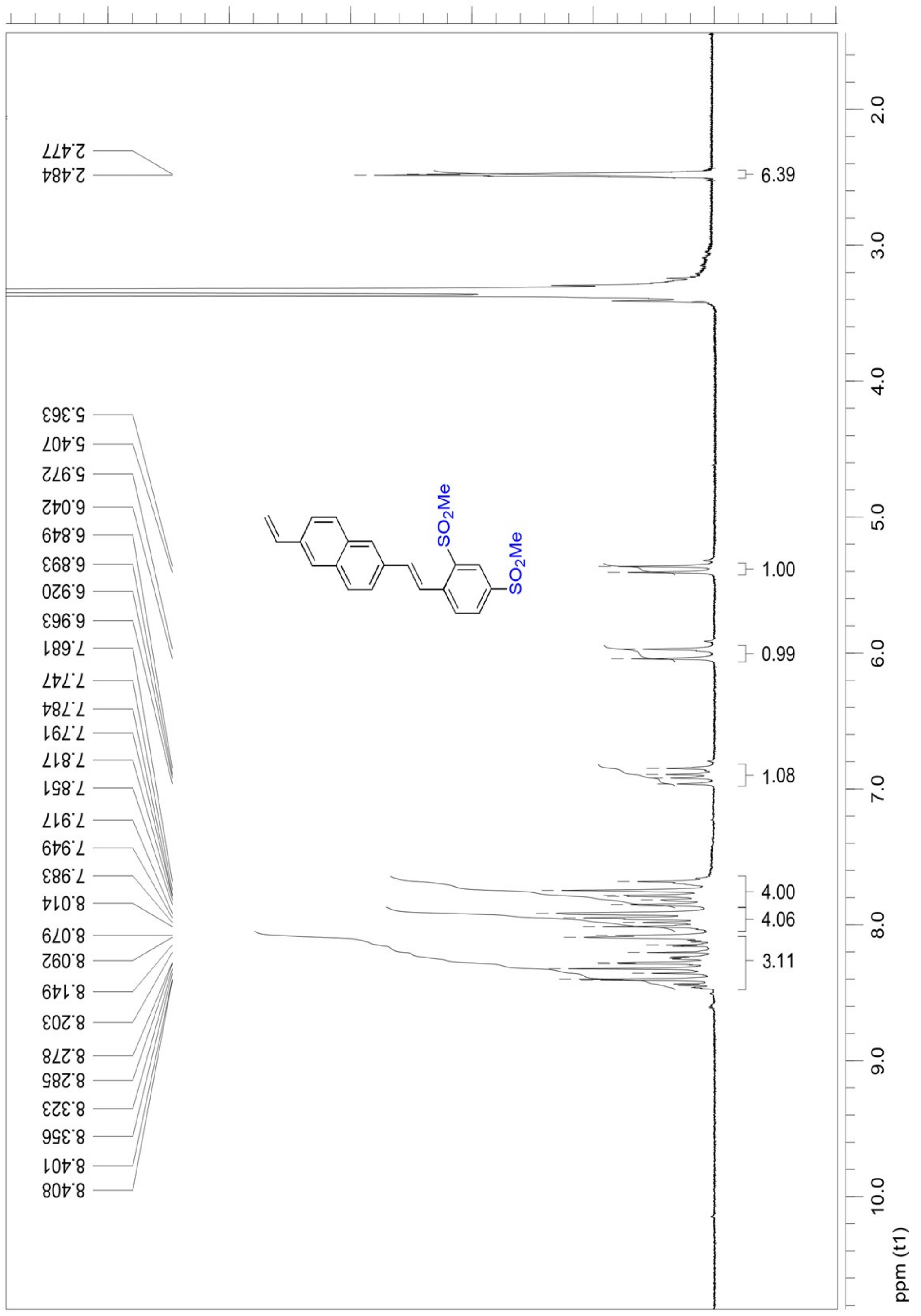

^{13}C NMR of 2-[2-(2,4-Bis-methanesulfonyl-phenyl)-vinyl]-6-vinyl-naphthalene

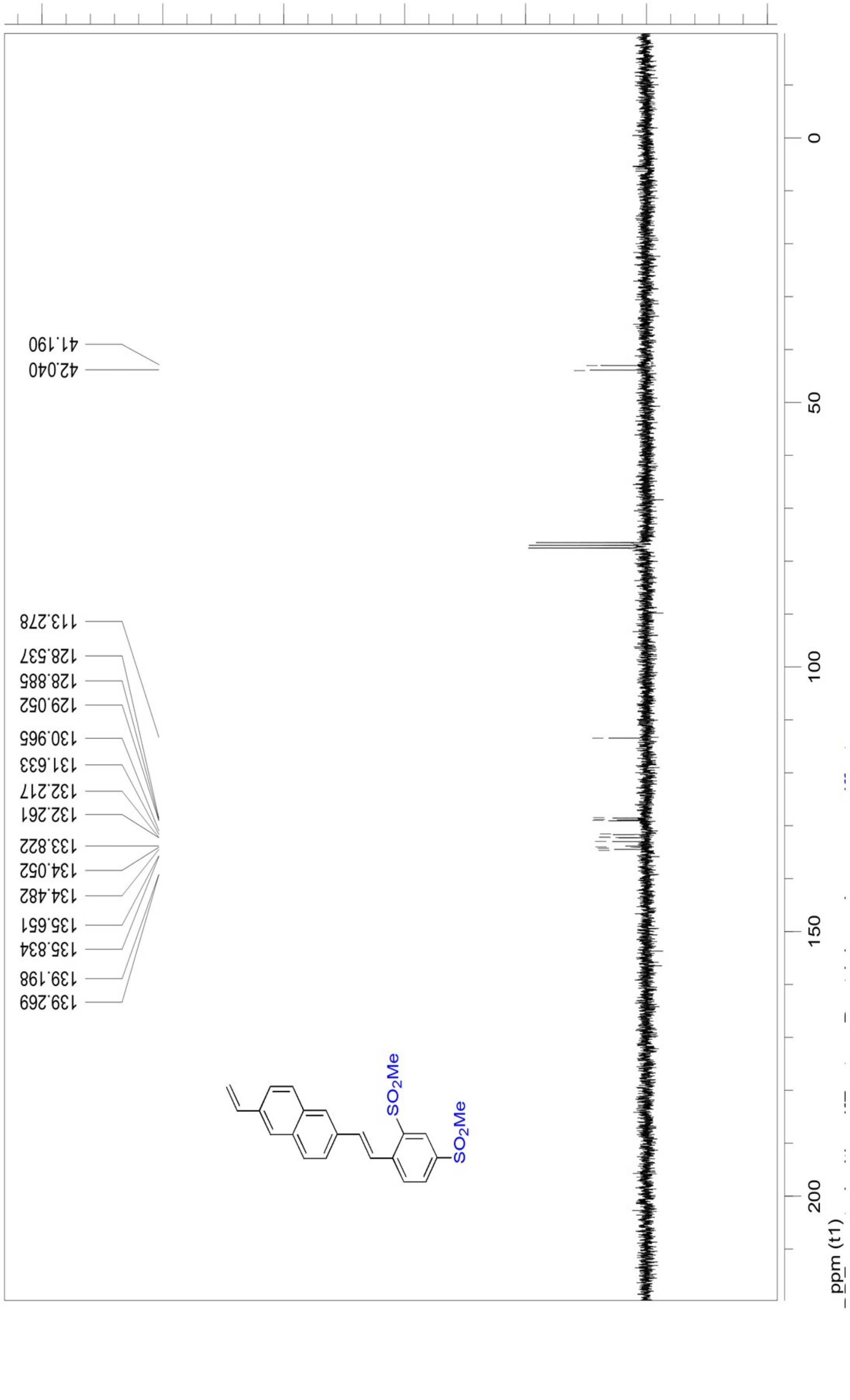

^{1}H NMR of 2-{[4-(2-{6-[2-(2,4-Bis-methanesulfonyl-phenyl)-vinyl]-naphthalen-2-yl}-vinyl)-phenyl]-ethyl-amino}-ethanol

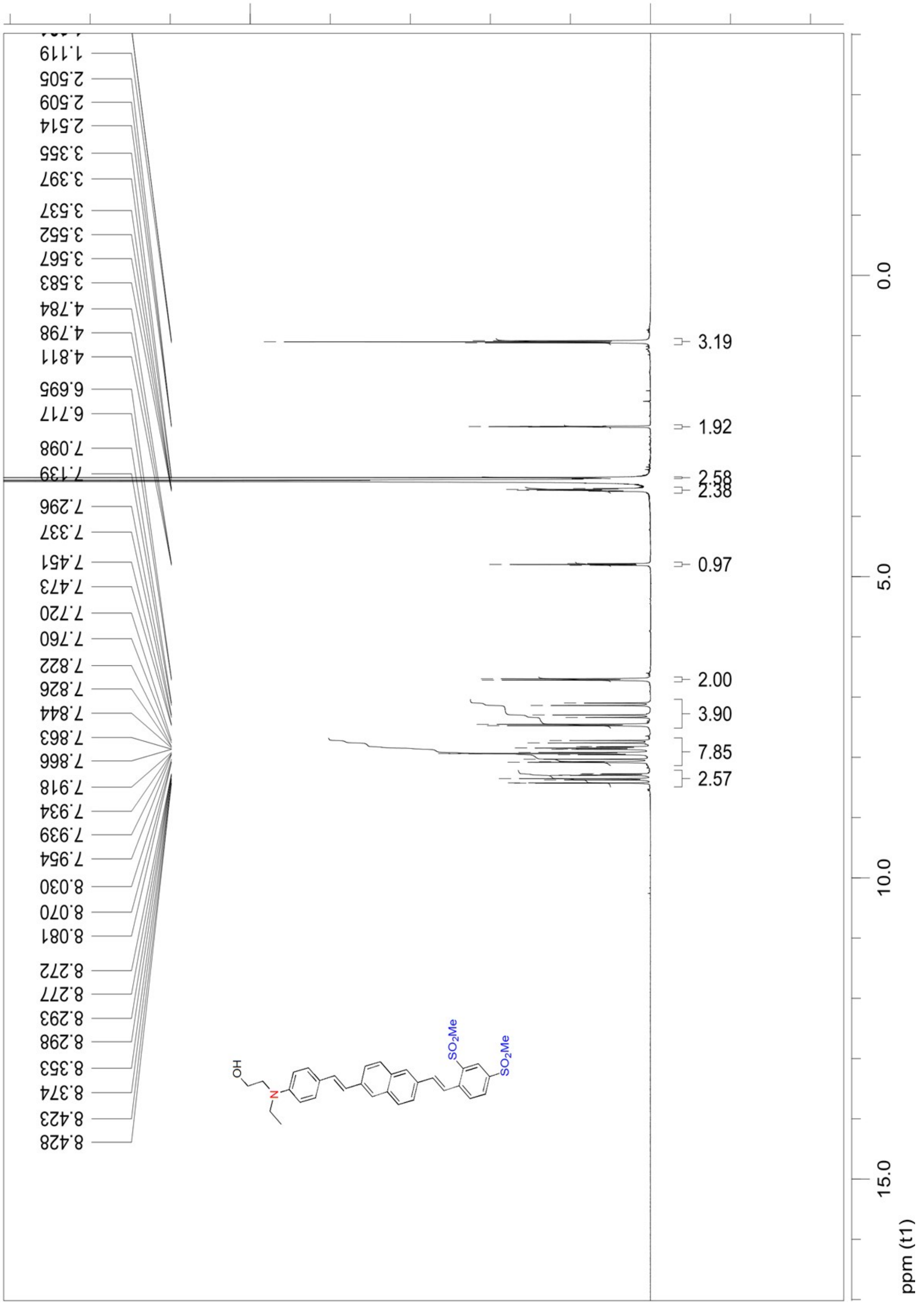

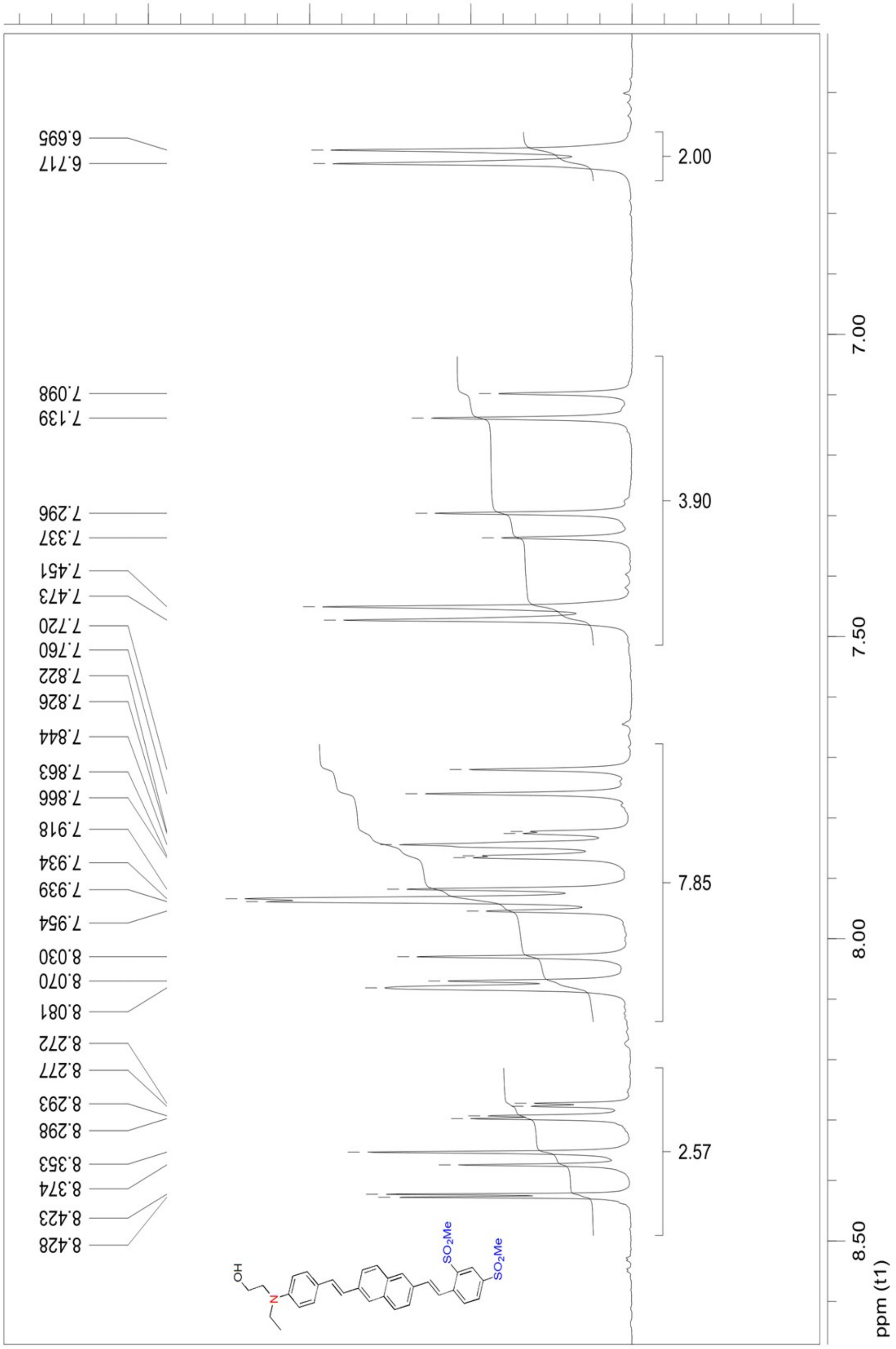
6.695
6.717
7.098
7.139
7.296
7.337
7.451
7.473
7.720
7.760
7.822
7.826
7.844
7.863
7.866
7.918
7.934
7.939
7.954
8.030
8.070
8.081
8.272
8.277
8.293
8.298
8.353
8.374
8.423
8.428
2.00
3.90
7.85
2.57
7.00
7.50
8.00
8.50
ppm (t1)
OH
N
SO2Me
SO2Me

^{13}C NMR of 2-{[4-(2-{6-[2-(2,4-Bis-methanesulfonyl-phenyl)-vinyl]-naphthalen-2-yl}-vinyl)-phenyl]-ethyl-amino}-ethanol

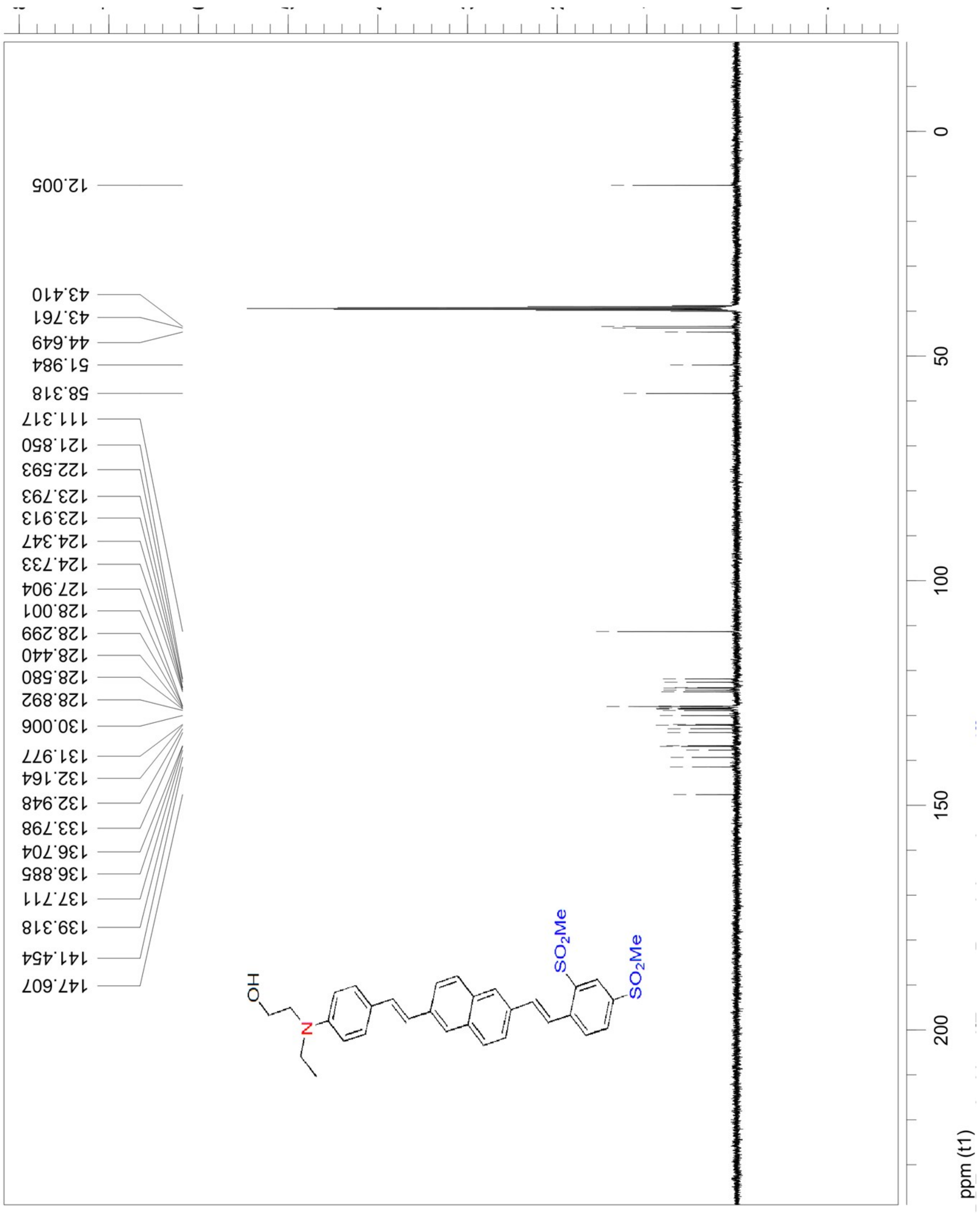

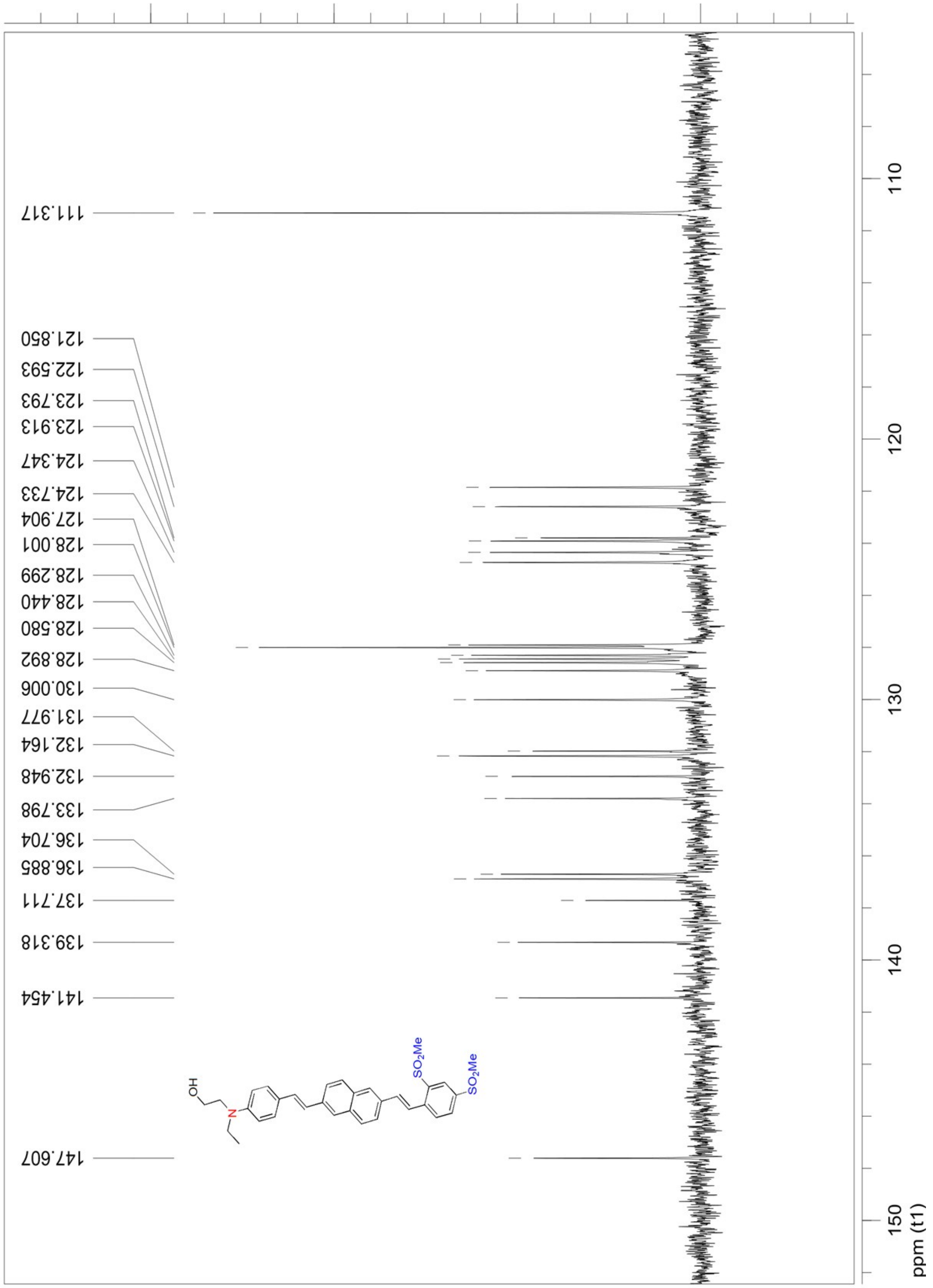
111.317
121.850
122.593
123.793
123.913
124.347
124.733
127.904
128.001
128.299
128.440
128.580
128.892
130.006
131.977
132.164
132.948
133.798
136.704
136.885
137.711
139.318
141.454
147.607
110
120
130
140
150
ppm (t1)
SO2Me
SO2Me
OH
N
